# Soluble Fas and the −670 Polymorphism of Fas in Lupus Nephritis

**DOI:** 10.1155/2014/780406

**Published:** 2014-11-18

**Authors:** Juan José Bollain-y-Goytia, Mariela Arellano-Rodríguez, Felipe de Jesús Torres-Del-Muro, Leonel Daza-Benítez, José Francisco Muñoz-Valle, Esperanza Avalos-Díaz, Rafael Herrera-Esparza

**Affiliations:** ^1^Laboratorios de Inmunología y Biología Molecular, UA Ciencias Biológicas, Universidad Autónoma de Zacatecas, 98040 Zacatecas, ZAC, Mexico; ^2^Unidad Médica de Alta Especialidad (UMAE) HPG No. 48, Instituto Mexicano del Seguro Social (IMSS), 37320 León, GTO, Mexico; ^3^Instituto de Investigación en Ciencias Biomédicas, Centro Universitario de Ciencias de la Salud, Universidad de Guadalajara, 45178 Guadalajara, JAL, Mexico

## Abstract

This study was performed to clarify the role of soluble Fas (sFas) in lupus nephritis (LN) and establish a potential relationship between LN and the −670 polymorphism of Fas in 67 patients with systemic lupus erythematosus (SLE), including a subset of 24 LN patients with proteinuria. Additionally, a group of 54 healthy subjects (HS) was included. The allelic frequency of the −670 polymorphism of Fas was determined using PCR-RFLP analysis, and sFas levels were assessed by ELISA. Additionally, the WT-1 protein level in urine was measured. The Fas receptor was determined in biopsies by immunohistochemistry (IHC) and *in situ* hybridization (FISH) and apoptotic features by TUNEL. *Results*. The −670 Fas polymorphism showed that the G allele was associated with increased SLE susceptibility, with an odds ratio (OR) of 1.86. The sFas was significantly higher in LN patients with the G/G genotype, and this subgroup exhibited correlations between the sFas level and proteinuria and increased urinary WT-1 levels. LN group shows increased expression of Fas and apoptotic features. In conclusion, our results indicate that the G allele of the −670 polymorphism of Fas is associated with genetic susceptibility in SLE patients with elevated levels of sFas in LN with proteinuria.

## 1. Introduction

Systemic lupus erythematosus (SLE) is a systemic autoimmune disease characterized by the production of autoantibodies and multiorgan involvement, including kidney damage in 60% of patients [1, 2]. Antinuclear antibodies (ANAs) are the hallmark of SLE, and specific anti-Sm, anti-dsDNA, anti-nucleosome, anti-C1q, and anti-GBM antibodies have been associated with LN [3–5].

Renal involvement is a serious complication of SLE because it can lead to high rates of morbidity and mortality [6]. The diagnosis of glomerulonephritis is suspected when proteinuria and urinary sediment alteration are accompanied by arterial hypertension. These data may predict kidney involvement, although renal biopsy remains the gold standard for the diagnosis and classification of LN. In particular, histological analysis can be used to identify the lesion and progression stages of renal disease according to the activity and chronicity index. However, despite the benefits of renal biopsy, this is an invasive procedure that requires an exhaustive review by a skilled pathologist; therefore, alternative biomarkers to identify renal disease are urgently needed [7]. Currently and traditionally used urinary biomarkers include proteinuria >0.5 g/L, alterations in renal ultrasound results, and changes in the rate of glomerular filtration, indicating the degree of renal function. Recently, additional urinary biomarkers have been used to predict renal damage, including markers of urinary podocytes, such as the transcription factor Wilm's tumor 1 (WT-1) [8].

Genetic susceptibility to SLE involves certain major histocompatibility complex class II (MHC II) alleles, such as HLA-DRB1^*^0301 and DRB1^*^1501 [9]. In addition, polymorphisms in genes encoding the cytokines interleukin-10 (IL-10), IL-6, tumor necrosis factor-*α* (TNF-*α*), and interferon-*γ* (IFN-*γ*) have been associated with SLE. Therefore, these polymorphisms could confer different degrees of susceptibility according the ethnic group [10–12]. Additionally, mutations in cytokine receptors and costimulatory molecules, such as CD28/B7 [13], and polymorphisms in genes associated with apoptosis, such as Fas, FasL, and Bcl-2, have been implicated in disease pathogenesis [14]. Accordingly, SLE is a polygenic disease in which different genes may be associated with different SLE disease subsets, including the nephritogenic phenotype.

Polymorphisms in the GMCP-1 gene were previously shown to be associated with increased susceptibility to SLE in a Mexican mestizo population [15, 16]. Additionally, the −1149 G/T polymorphism of the prolactin promoter has been correlated with the production of anti-DNA antibodies [17]. In contrast, the −653 G/A NRF-2 (erythroid nuclear factor-2) polymorphism does not increase SLE susceptibility during childhood, although such polymorphisms may be associated with LN [18].

The role of apoptosis in SLE has been intensively studied, and the Fas/CD95/Apo-1 gene has been mapped to the 10q 24.1 region. This gene consists of nine exons and eight introns as well as the promoter responsible for allelic variations in Fas, which can also modify the transcriptional rate. For example, if a guanine (G) is replaced by an adenine (A) at position −670, the resulting polymorphism increases the binding affinity of the transcription factor STAT-1 for the interferon gamma-activated sequence (GAS), which in turn alters the transcription rate of the Fas receptor [19]. Another polymorphism of Fas at position 297, with the presence of a C allele, is associated with SLE development in the Japanese population; interestingly, this polymorphism does not increase the risk for SLE in the Italian population [20].

The Fas receptor exists in two forms; one form is anchored to the plasma membrane, whereas the other is soluble (sFas). The latter form is highly regulated at the transcriptional level [21, 22]. sFas plays a role as an antiapoptotic molecule that blocks FasL or sFasL binding, and its concentration in the serum of healthy subjects is independent of gender and age [23]. Additionally, in clinical practice, sFas has been defined as a marker of inflammation related to endothelial dysfunction in chronic renal diseases [24–26]. In SLE, sFas levels are increased due to a deletion in exon 6 [21, 23, 27], although knowledge about its participation in LN is lacking.

The present study was performed to assess the possible role of the −670 Fas polymorphism in LN and address the issue of whether increased levels of sFas are related to podocyte damage, proteinuria, and autoantibody production.

## 2. Materials and Methods

### 2.1. Biological Samples

This cross-sectional study of cases and controls analyzed the −670 polymorphism of Fas in a mestizo group of SLE patients living in the north-central region of Mexico. The mean age of the subjects was 41.2 ± 22.1 years, and all patients met the American College of Rheumatology (ACR) criteria for SLE classification [28] (females 79.6% and males 20.3%).

The SLE group was divided into two subsets. This first group lacked evidence of renal involvement, tested positive for ANAs (80%) or anti-dsDNA antibodies (*Crithidia luciliae*) (7.14%), and showed negative or irrelevant levels of proteinuria (mean level of 0.116 g/L). The second group had LN with proteinuria levels higher than 0.5 g/L and displayed a positive ANA test result (90%) and a high prevalence of anti-dsDNA antibodies (67%). Additionally, a control group was included, consisting of 54 healthy subjects (HS) without evidence of autoimmune disease. This group was 84.31% female and 15.6% male with an average age of 34.3 ± 14.51 years, and all HS tested negative for ANAs, anti-dsDNA antibodies, and proteinuria. This study was performed according to the principles of the Declaration of Helsinki and was approved by the ethics committees of our institutions. After providing detailed information, signed informed consent was obtained from the patients and controls.

### 2.2. Blood Collection

Peripheral blood was collected in Vacutainer 7.2 mg K2 EDTA tubes and used for DNA extraction; simultaneously, tubes without anticoagulant were used to obtain serum.

### 2.3. Autoantibodies

ANA measurements were performed by immunofluorescence (IF) using HEp-2 cells and anti-DNA antibodies by* Crithidia luciliae* (Immuno Concepts NA, Ltd., Sacramento, CA). The following antibody specificities were quantified by enzyme-linked immunosorbent assay (ELISA): anti-Ro-60 (EA 1595-9601 G), anti-La-48 (EA 1597-9601 G), anti-nRNP/Sm (EA 1591-9601 G), and antiglomerular basement membrane (GBM) (EA 1251-9601 G), according to the manufacturer's recommendations (Euroimmun US Inc.).

### 2.4. Soluble Fas

sFas levels were determined using a commercial ELISA kit (Quantikine Human sFAS/TNFRSF6 R&D System, Abingdon, UK), and the optical density (OD) was measured at 450 nm using an ELISA plate reader (Multiskan FC, Thermo Scientific). The sFas concentration was expressed in *ρ*g/mL according to the curves obtained from the standards.

### 2.5. Restriction Fragment Length Polymorphism (PCR-RFLP) Analysis

The −670 polymorphism of the Fas receptor was analyzed using PCR-RFLP analysis, as previously reported [19]. DNA from peripheral blood was extracted using Miller's modified technique [29]. The PCR reaction was performed using Taq DNA polymerase (Platinum High Fidelity from Invitrogen, Life Technologies) as follows. First, 1 *μ*g of DNA was placed in an Eppendorf tube with a reaction mixture containing 0.2 mM of the sense (5′-CTACCTAAGAGCTATCTACCGTTC-3′) and antisense (5′-GGCTGTCCATGTTGTTGGCTGC-3′) oligonucleotides; then, 5 *μ*L of 10X high fidelity PCR buffer, 25 *μ*L of 2X nucleotides, 2 *μ*L of 50 mM MgSO_4_, and 0.2 *μ*L of Taq enzyme mixture were incubated at 4°C and adjusted to a final volume of 50 *μ*L with H_2_O (GIBCO Ultrapure). The PCR reaction was performed in a Perkin Elmer 2400 thermocycler using 35 cycles at the following conditions: 94°C for 2 min, 94°C for 30 s, 58°C for 30 s, and 72°C for 30 s. A final reaction extension was performed at 72°C for 10 min. PCR products were digested at 37°C for 1 h with the restriction enzyme Mva-I (Cat. number 11288075001, Roche Diagnostics, Indianapolis, USA), and 10 *μ*L of digested and undigested PCR products was separately run in a 2% agarose gels using 1X TAE buffer for 40 min at 80 volts; the gels were then stained with ethidium bromide. A DNA ladder was used (1 Kb Plus DNA Ladder of Invitrogen), and the gels were analyzed using the Carestream Molecular Imaging Software, version 5.0.

### 2.6. Fas Expression Was Determined in Renal Biopsies by Immunohistochemistry (IHC) and by* In Situ* Hybridization (FISH)

#### 2.6.1. Preparation of Fas Fluorescent Probes

Fluorescent-labelled PCR-derived probes were synthesized by PCR using a random-primed *λ*gt11-human spleen library as a template (Clontech, Palo Alto, CA, USA), using the following oligonucleotides: Fas forward 5′-GGT GGG TTA CAC TGG TTT ACA-3′ and backward 5′-GTG CTA CTC CTA ACT GTG AC-3′ [30]. The PCR reaction was done by incubation of 1 *μ*g of template with 25 *μ*M of nucleotides 2X dNTP, 100 *μ*M fluoro-Red-labelled-UTP (Amersham Biosciences, Buckingham, UK), and 0.2 *μ*M of the aforementioned primers mixed with 0.5 U/50 *μ*L of DNA polymerase (Platinum Taq DNA polymerase High Fidelity of Invitrogen Life Technology Ltd, Carlsbad, CA USA). The reaction tubes containing 50 *μ*L of the sample mixture were amplified in a thermocycler (PerkinElmer, GeneAmp PCR system 2400) using 30 cycles (94°C for 2 min, 48°C for 2 min, and 72°C for 1.4 min). At the end of the PCR, the amplificates were electrophoresed in 0.8% agarose. The internal fluorescent red labeling of the PCR products was observed under ultraviolet light as reed bands in agarose gels lacking in ethidium bromide.

#### 2.6.2. *In Situ* Hybridization

Slides containing 4 *μ*m sections of renal tissues were incubated with 0.02 M HCl, permeabilized with 0.01% Triton X-100/PBS-DEPC, and washed in cold 20% acetic acid. Probes were adjusted to 50 ng/mL in 1 : 1 hybridization buffer: formamide and were applied individually to the tissues. Tissues were prehybridized at 90°C for 3 min, followed by hybridization at 37°C for 24 hrs, and were washed in SSC 2X buffer. In addition, following the washes some slides were counterstained with 4′,6-Diamidine-2′-phenylindole dihydrochloride (DAPI) [31]. Finally, the slides were mounted and evaluated under confocal microscope. The intensity of the color red obtained by FISH was analyzed in the software Image-Pro Plus Versión 7.0. (Media Cybernetics, USA).

### 2.7. Biopsies

Tissues were from patients with LN and control biopsies obtained during necropsy of individuals who died in a car accident, after obtaining written consent from their families. In all the patients, kidney biopsies were performed percutaneously, and a segment of each biopsy was stained for hematoxylin and eosin (H and E) and evaluated under light microscopy. The biopsies were classified according to the ISN/RPS 2004 classification of LN [32].

### 2.8. Immunohistochemistry

The Fas receptor was detected by IHC on 4 *μ*m thick sections of renal tissues mounted on microscope slides. The specimens were dewaxed, permeabilized with 0.01% Triton X-100/phosphate buffered saline (PBS), and then washed thrice with PBS. Endogenous peroxidase was blocked for 10 minutes with 3% H_2_O_2_ dissolved in methanol. After an additional wash, the tissues were incubated for 12 hours with a monoclonal anti-APO-1 (DAKO) and diluted 1 : 100 in 10% FBS-PBS, the tissues were then washed in several changes of PBS, and the bound antibodies were identified with HRP-goat anti-mouse IgG (Zymed, Laboratories Inc., San Francisco, CA). The color reaction was induced by 3,3′-diaminobenzidine-0.06% H_2_O_2_ (Sigma, St. Louis, MO), and the reaction was stopped with 0.5 M sulfuric acid. The slides were then examined under a light microscope. The assays were performed in triplicate and evaluated by two pathologists in a blinded fashion. The intensity of the color reaction obtained by IHC was analyzed in the software Image-Pro Plus Versión 7.0. (Media Cybernetics, USA).

### 2.9. Other Parameters

Proteinuria was measured using the conventional dry chemistry method. The level of the WT-1 podocyte marker was measured by ELISA in urine collected over a 24 h period using a previously described method [8]. Apoptotic features were detected by TUNEL (Roche Molecular Biochemical's. Penzberg, Germany).

### 2.10. Statistical Analysis

Differences in the measured parameters between different groups were evaluated by analysis of variance (ANOVA) tests with Tukey's and Pearson's correlations. GraphPad Prism version 17 was used for analysis, and *P* values < 0.05 were considered significant.

## 3. Results

### 3.1. The −670 Fas Polymorphism in SLE

**Figure 1:**
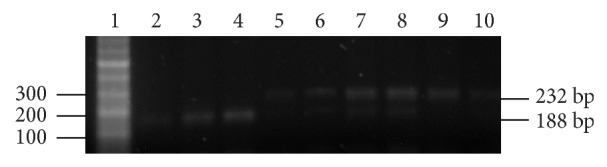
Electrophoresis of PCR products in patients and controls. The polymorphic fragments A/A resulted in a 232 bp band, the G/G fragment resulted in a 188 bp band, and the A/G variation resulted in a doublet of the 188 bp and 232 bp bands on a 2% agarose gel.

After the PCR reaction, the products were digested to obtain the polymorphic fragments. The A/A genotype was identified as a 232-base pair (bp) band, the G/G genotype appeared as a 188 bp band, and the heterozygous A/G variant appeared as a doublet of the 188 bp and 232 bp bands, as shown in Figure 1. The A and G allelic frequencies were 0.41 and 0.45, respectively. These results indicated that the Fas G allele was associated with susceptibility to SLE, with odds ratios (ORs) of 1.86 (*P* = 0.03) and 2.23 (*P* = 0.05) for the dominant and recessive models, respectively (Table 1).

### 3.2. Soluble Fas Is Increased in Lupus Nephritis

**Figure 2:**
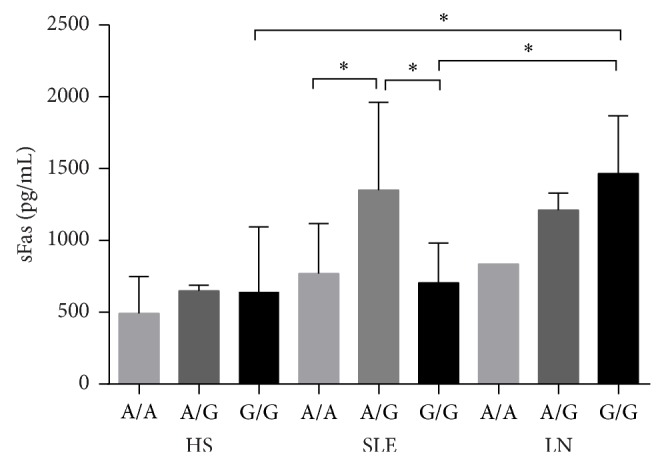
The sFas concentration according to −670 Fas genotype in LN and SLE patients and HS. The concentration of sFas was increased in LN patients with the G/G genotype and was significantly different from patients with SLE. ^*^ *P* < 0.05.

The sFas level was slightly elevated in the LN subset with the G/G genotype compared to SLE patients without LN (Figure 2).

### 3.3. Association between sFas and the −670 Fas Polymorphism

**Figure 3:**
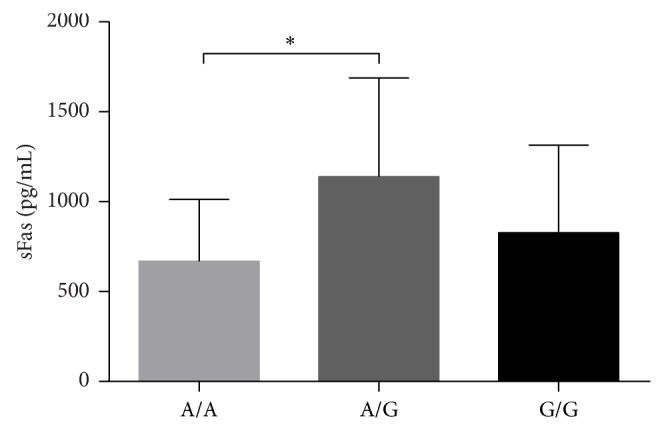
sFas concentration according to −670 Fas genotype. The sFas concentrations in patients with the A/A and G/G genotypes were not significantly different. In contrast, the sFas concentration in patients with the A/G genotype was different compared to other genotypes. ^*^ *P* < 0.05.

To address the question of whether sFas is associated with the −670 polymorphism of the Fas receptor, the sFas levels in the serum were compared between the A/A, A/G, and G/G genotypes. The average concentration of sFas for the A/A genotype was 668.99 ± 344.04 *ρ*g/mL, whereas the concentration for the A/G genotype was 1,140.17 ± 559.89 *ρ*g/mL, and this difference between genotypes was significant (*P* < 0.05). In contrast, the G/G genotype showed a mean concentration of 828.06 ± 486.78 *ρ*g/mL, and this value was not significantly different compared to the A/A or A/G genotype (Figure 3).

### 3.4. sFas Is Increased and Correlated with Autoantibodies and Proteinuria

**Figure 4:**
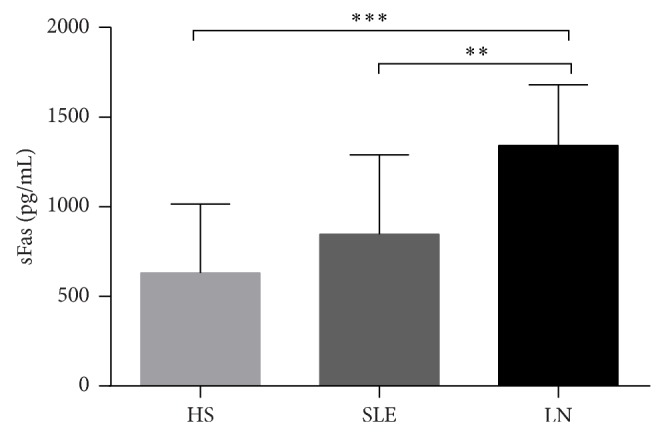
The sFas level in the serum of SLE and LN patients and HS. The concentration of sFas was increased in patients with LN. ^**^ *P* < 0.01; ^***^ *P* < 001.

The concentration of sFas was increased regardless of the age of the SLE patients, with a mean value of 845.84 ± 444.66 *ρ*g/mL, whereas this concentration was 1,342.997 ± 337.10 *ρ*g/mL in LN subjects. When these values were compared to those of the HS (630.44 ± 385.34 *ρ*g/mL), there was a significant difference (*P* < 0.001) (Figure 4).

### 3.5. Fas Protein and mRNA Is Expressed in Glomerulus

**Figure 5:**
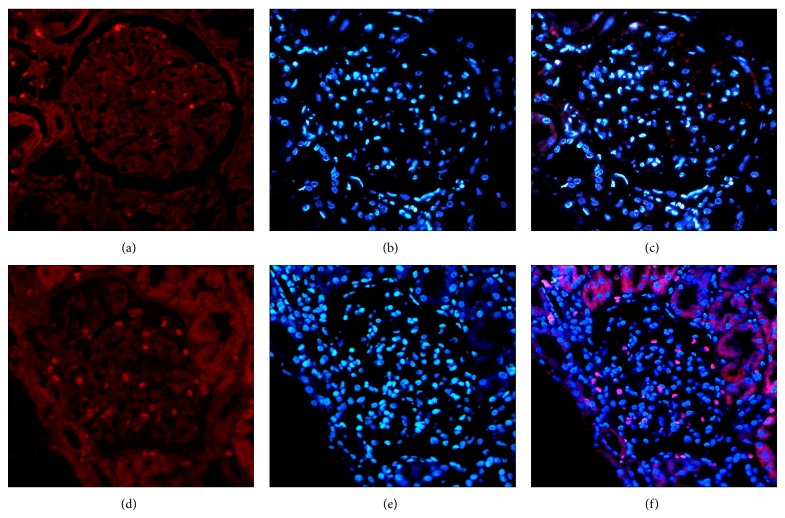
The expression of mRNA Fas receptor in renal tissue of LN patients and HS by FISH. The upper panel (a), (b), and (c) shows the HS tissue, and the lower panel (d), (e), and (f) shows a representative LN. (a) and (d) Expression of the mRNA Fas receptor (red). (b) and (e) Staining in blue by DAPI. (c) and (f) show the overlapping (pink).

**Figure 6:**
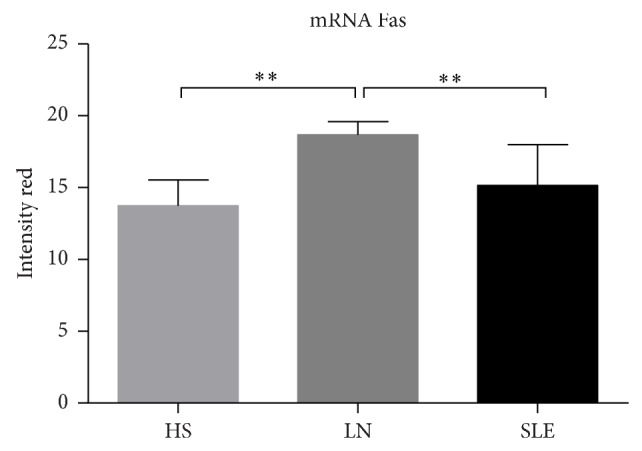
Expression the mRNA Fas receptor in renal tissue of SLE, LN patients, and HS. Difference between LN with other groups was significant. ^**^ *P* < 0.006.

The lupus nephritis biopsies included were 14 that had Class IV and 10 with Class III. Patients with Class IV nephritis displayed the highest activity and chronicity scores, while control kidneys had no histological evidence of renal disease (Figures 5 and 6).

### 3.6. The Fas Protein Expression Was Increased in Lupus Nephritis

**Figure 7:**
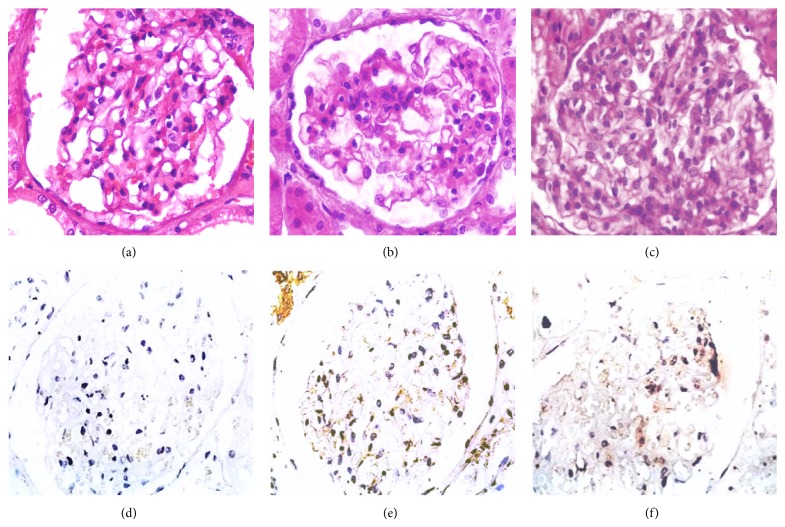
The expression of Fas receptor in renal tissue of LN patients and HS by IHC. Superior panel stained by H and E. Inferior panel, IHC for Fas. (a) and (d) HS biopsies; (b), (c), (e), and (f) show a representative of LN. (b) and (e) LN Class-IV. (c) and (f) LN Class-III.

**Figure 8:**
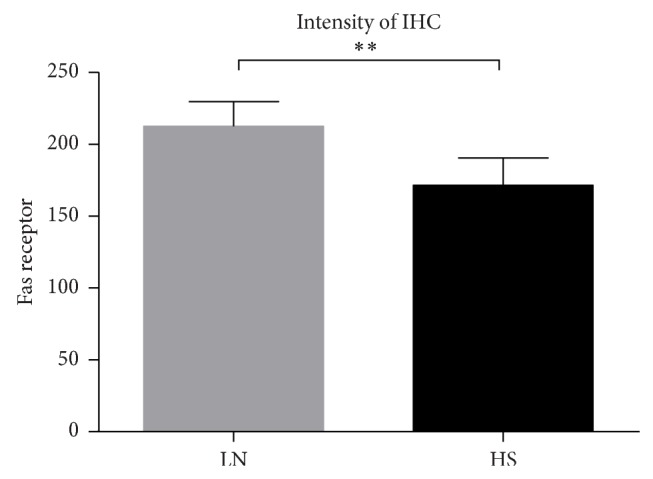
Expression of Fas receptor in renal tissue of LN patients and HS. The expression of Fas was increased in patients with LN (^**^ *P* < 0.007).

The LN biopsies broadly expressed the Fas receptor, which was mainly detected along the tubules, in glomerular endothelial cells and in the mesangium. Although HS biopsies showed similar distributions of Fas, their staining intensities were lower (Figure 7). Additionally, the color intensity was measured by an image analyzer program, and values were expressed as sum of intensities in pixels is one hundred fields. Using this approach, significant differences of Fas between HS and LN were observed (Figure 8).

### 3.7. Apoptotic Cell in Glomeruli

**Figure 9:**
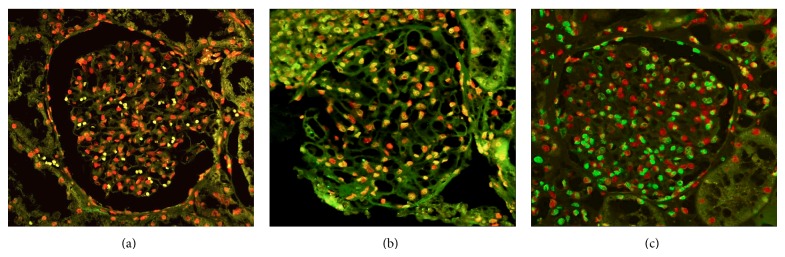
TUNEL assays in renal tissue of LN patients and HS. Positive apoptotic cell exhibited nuclei tagged in green. (a) HS biopsies. (b) and (c) show a representative of LN. The nuclei of nonapoptotic cell were red-tagged.

An increase in values obtained by apoptotic cells in LN was observed, and significant differences between HS and LN were observed (Figure 9 and Table 2).

**Figure 10:**
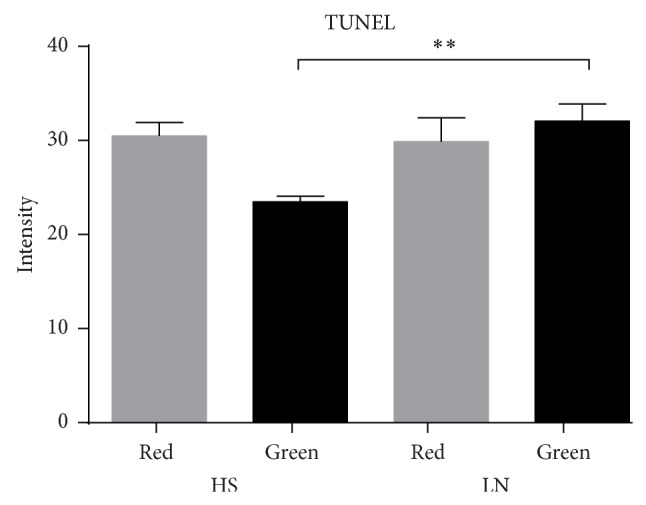
TUNEL assays in renal tissue of LN patients and HS. The intensity green belongs to glomerular apoptotic cells in LN biopsies, the red color belongs to nonapoptotic cells. ^**^ *P* < 0.008.

Additionally, there was a significant difference between the SLE and LN subsets (*P* < 0.01) (Figure 10). LN subjects demonstrated a positive ANA test (90%) and a high prevalence of anti-dsDNA antibodies (67%); this result was in contrast with the SLE group without nephritis, which showed an ANA level of 80% with anti-dsDNA antibodies in only 7.14% of cases. Thus, we next sought to determine whether sFas in LN is associated with a high prevalence of autoantibodies, including anti-DNA antibodies, and proteinuria. To this end, a Pearson's correlation analysis was performed, and as expected, a significant correlation among proteinuria, podocyturia (as measured by WT-1), and the levels of anti-dsDNA and anti-Ro-60 antibodies was observed in the LN subset. However, there was a lack of correlation with anti-nRNP/Sm and anti-La-48 autoantibodies (Table 3).

## 4. Discussion

In the present investigation, the −670 polymorphism of the promoter region of the Fas gene was analyzed. We also sought to determine whether the association between LN and sFas levels is associated with the −670 Fas polymorphism. Finally, we evaluated whether these factors are associated with the lupus nephritis susceptibility in the Mexican mestizo population.

The present results suggest that the G allele of the Fas promoter is associated with SLE susceptibility (OR, 1.86). Second, an increased serum level of sFas was detected in LN patients with the G/G and A/G −670 genotypes. Third, this increase in sFas among LN patients with proteinuria and podocyturia, suggests that the increase in the sFas level may be transcriptionally regulated, and it is associated to the G/G and A/G genotypes. Fourth, the broad expression of Fas receptor in LN biopsies correlated with apoptotic features.

Associations between the A/G −670 Fas polymorphism and autoimmune diseases, such as type 1 diabetes, multiple sclerosis, Sjögren's syndrome, rheumatoid arthritis (RA), and SLE, have been described [19, 33, 34, 46]. Nevertheless, it remains unclear how this polymorphism participates in LN pathogenesis.

The present results suggest that the genotype and allelic frequency of the G allele of the Fas −670 polymorphism are associated with SLE in Mexican mestizo patients; therefore, this genotype may confer susceptibility to SLE. We should note that the studied population lives in the north-central area of Mexico, and our results differ with those reported in Japanese SLE patients in which the A allele was associated with SLE development [43]. However, these results suggest that the Fas promoter partially contributes to the pathogenesis of SLE [33]. Other studies in India have reported the association between SLE and the −670 Fas polymorphism [37], and the allelic differences with the present work seem to be due to the mixed ethnic groups present in the Mexican mestizo population [47].

Our results agree with other reports showing that the −670 polymorphism is associated with certain ANA specificities, which is in agreement with a report on Korean SLE patients [44]. Taking into account the fact that different genes participate in SLE, transcriptional regulation of the Fas receptor in SLE seems to be activated by IFN-*γ* [43].

Regarding the possible association between the −670 polymorphism and sFas levels, we found that sFas levels were increased in LN patients with the A/G and G/G genotypes, which is in agreement with previous reports [33, 37, 43].

The meta-analysis studies related with Fas −670 A/G polymorphism show that such genotype confers susceptibility to SLE in Asian population (Table 4) [33, 34]. We should note that these studies do not include any Latin America country; this is the first study on the Fas −670 polymorphism in a population of north-central area of Mexico, as depicted in Table 4, that compares our results with other reports [33, 34, 37, 40, 43, 44, 47]. The studies on the association between Fas −670 A/G polymorphism and SLE produced controversial results; it may be because of the clinical heterogeneity, different ethnicities, and real genetic heterogeneity. Another possible explanation is the small sample size; nevertheless our results agree with other reports showing the association between the Fas −670 G allele carrier and SLE [33]. The association of functional polymorphisms in the promoter of Fas with SLE susceptibility has been a controversial issue. Therefore different single-nucleotide polymorphisms have been identified in the promoter region of Fas; one of them is the substitution of A to G at position −670, which theoretically affect the binding ability of the GAS binding protein to the nuclear transcription element STAT-1; this genotype decreases the promoter activity and consequently the Fas-expression. Regarding the two forms of Fas receptor, we should note that both normal Fas and the sFas transcripts are derived by the same gene promoter; in the case of sFas transcript it results from an alternative splicing that truncates the transcript and results in a protein that lacks intracellular and transmembrane domains (sFas). In theory the increase of the sFas level might antagonize the Fas-FasL apoptotic pathway [48]; nevertheless and taking into account our results, the mRNA of Fas as well as the Fas protein was fully expressed at glomerular level as our FISH and immunohistochemistry assays demonstrated; furthermore the Fas pathway was functional because we were able to demonstrate correlation between Fas receptor expression and apoptotic features of lupus nephritis patients as the TUNEL assays demonstrated; another alternative explanation for sFas increasing in LN patients could be secondary to the local inflammatory process. In this scenario the matrix metalloproteinases (MMP) are produced and are related with renal dysfunction; therefore MMP associates to proliferating events at glomerular level, and therefore enhanced MMP activity in lupus nephritis patients as well as in experimental models has been reported [49], interestingly MMP can digest part of the extracellular domain of Fas receptor [26] increasing the sFas levels; it might be the case of our findings in lupus nephritis patients, as we observed in patients with active renal disease, and previously the association of high levels of sFas in patients with kidney damage by lupus was reported [35, 50, 51].

Additionally, another study related with Fas −1377 polymorphism in SLE patients shows an increase in the rate of Fas transcription, which may increase the number of apoptotic cells, resulting in a deficiency in the clearing of apoptotic bodies. These cells undergo secondary necrosis, releasing intracellular antigens that break down immune tolerance, resulting in autoimmunity and tissue damage that affects natural filters, such as the kidneys. In the present study, we demonstrate that increased levels of sFas correlate with the presence of autoantibodies and proteinuria due to podocyte damage, and our results confirm previous reports [25].

Finally, we observed a higher sFas concentration in SLE patients with greater proteinuria, reflecting podocyte damage according to the increased levels of the urinary biomarker WT-1. As a result, sFas may be used as an alternate biomarker in patients with LN, as these levels may predict damage to the ultrafiltration glomerular unit. In conclusion, the present study indicates that the G allele of the Fas −670 polymorphism is associated with genetic susceptibility to SLE as well as increased levels of sFas in patients with LN.

## Figures and Tables

**Table 1:** Genotype and alleles frequencies of the −670 A/G Fas polymorphism in SLE and LN patients and HS.

	SLE (*n* = 43) % (*n*)	LN (*n* = 24) % (*n*)	HS (*n* = 54) % (*n*)	*P* value
Genotype				
A/A	33 (14)	29 (7)	52 (28)	^§,&^ *P* = 0.22
A/G	30 (13)	38 (9)	22 (12)	
G/G	37 (16)	33 (8)	26 (14)	
Allele				
A	48 (41)	48 (23)	63 (68)^§^	^§,&^ *P* = 0.03^*^
G	52 (45)^&^	52 (25)	37 (40)	OR = 1.86 95% CI = 1.007–3.45
**A/A** + **A**/G	63 (27)	67 (16)	74 (40)	
GG	37 (16)	33 (8)	26 (14)	*P* = 0.23
AA	33 (14)	29 (7)	52 (28)^Φ^	*P*= 0.05^*^
A/**G** + **G/G**	67 (29)^*φ*^	71 (17)	48 (26)	OR = 2.23 95% CI = 0.90–5.6

LN: lupus nephritis; SLE: systemic lupus erythematosus; HS: healthy subjects; ^§^HS versus SLE; ^&^SLE versus HS; ^Φ^HS versus SLE; ^*φ*^SLE versus HS. ^*^A *P* value <0.05 is significant.

**Table 2:** Biomarkers in HS and lupus nephritis.

Group	HS	LN	*P* value
sFas *ρ*g/mL	630.44 ± 385.34	1,342.99 ± 337.10	0.001
mRNA Fas	13.73 ± 1.805	18.68 ± 0.915	0.006
Protein Fas	171.51 ± 8.468	221.53 ± 7.642	0.007
Apoptotic cells	23.496 ± 1.283	32.059 ± 5.800	0.01

sFas: soluble Fas, HS: healthy subjects, LN: lupus nephritis. Significance *P* < 0.05.

**Table 3:** Correlation between sFas levels and markers of disease in LN patients.

	Proteinuria ≥ 0.5 g/L	WT-1	anti-Ro-60	anti-dsDNA	anti-MBG	nRNP/Sm	anti-La-48
sFas	*r* = **0.864**	*r* = **0.718**	*r* = **0.659**	*r* = **0.593**	*r* = 0.276	*r* = 0.522	*r* = −0.372
	*P* = 0.01^*^	*P* = 0.004^**^	*P* = 0.05^*^	*P* = 0.032^*^	*P* = 0.440	*P* = 0.150	*P* = 0.324

^*^Correlation is significant at the 0.05 level (two-tailed).

^**^Correlation is significant at the 0.01 level (two-tailed).

**Table 4:** Characteristics of the individual studies of the −670 (A/G) Fas polymorphism in SLE and sFAS adapted from [33, 34].

Studies	Years	Country	sFas levels *ρ*g/mL SLE/controls	Numbers SLE/controls	A alleles (%) SLE/controls	Association *P* value
Hatef et al. [35]	2013	Iran	409.38/168 *P* = 0.03	32/46	ND	ND
Moudi et al. [36]	2013	Iran	ND	106/149	58/49	0.03
Pradhan [37]	2012	India	4771.5/1131.4 *P* = ND	70/70	58/42	0.001
Molin et al. [38]	2012	Germany	ND	46/96	37/11.5	0.001
Man et al. [39]	2012	China	ND	552/718	61.1/58.1	NS
Araste et al. [40]	2010	Iran	158.1/48.7 *P* = 0.001	249/212	51.6/49.5	NS
Sahebari et al. [25]	2010	Iran	372.2/190.3 *P* = 0.001	114/50	ND	ND
Mahfoudh et al. [41]	2007	Tunisia	0/3,200	0/170	0/57	ND
Xu et al. [42]	2004	China	ND	103/110	NS	NS
Kanemitsu et al. [43]	2002	Japan	ND	109/140		0.004
Lee et al. [44]	2001	Korea	ND	87/86	NS	NS
Al-Maini et al. [45]	2000	United Arab Emirates	600/260 *P* = 0.0001	39/22	ND	ND
Huang et al. [19]	1997	Australia	ND	79/86	NS	NS
Jodo et al. [21]	1997	Japan	870/220 *P* = 0.001	77/40	ND	ND
Present study	2014	Mexico	1094.41/630.44 *P* = 0.001	67/54	48/63	0.03

sFas: soluble Fas, SLE: systemic lupus erythematosus, ND: undetermined, NS: not significant.
